# Supplementary material for: Highly selective electrocatalytic alkynol semi-hydrogenation for continuous production of alkenols
Source: Nat Commun. 2023 Mar 20;14:1533. doi: 10.1038/s41467-023-37251-z (PMC10027872; doi:10.1038/s41467-023-37251-z)
Supplement: Supplementary file 1 — Supplementary Information [file 41467_2023_37251_MOESM1_ESM.pdf]

Supplementary Materials for

Highly selective electrocatalytic alkynol semi-hydrogenation for continuous production of alkenols

Authors: Jun Bu[1,2], Siyu Chang[2], Jinjin Li[2], Sanyin Yang[2], Wenxiu Ma[2], Zhenpeng Liu[1], Siying An[2], Yanan Wang[3], Zhen Li[1], Jian Zhang[1,2*]

Affiliations:

[1] State Key Laboratory of Solidification Processing and School of Materials Science and Engineering, Northwestern Polytechnical University, Xi'an, Shaanxi, 710072, P. R. China.

[2] Key Laboratory of Special Functional and Smart Polymer Materials of Ministry of Industry and Information Technology and School of Chemistry and Chemical Engineering, Northwestern Polytechnical University, Xi'an, Shaanxi, 710129, P. R. China.

[3] Hualu Engineering and Technology Co., Ltd, Xi'an, Shaanxi, 710065, P. R. China.

*Corresponding author. Email: zhangjian@nwpu.edu.cn (J.Z.)

This PDF file includes:

The photograph of the used flow cell	2
Synthetic route of Cu nanoarrays	3
Morphology and structural characterization of Cu nanoarrays	4
The polarization curves of Cu nanoarrays with and without MBY	8
GC-MS analysis of the liquid products	9
Electrocatalytic MBY semi-hydrogenation performance of Cu nanoarrays	12
Characterization of Cu nanoarrays after stability test	18
Influence of MBY concentration on catalytic performance	22
Influence of electrolyte pH on catalytic performance	25
IR spectra of free MBY and MBE in liquid phase	29
Electrocatalytic performance over 25 cm^2 electrolyser	32

Supplementary figures and tables.

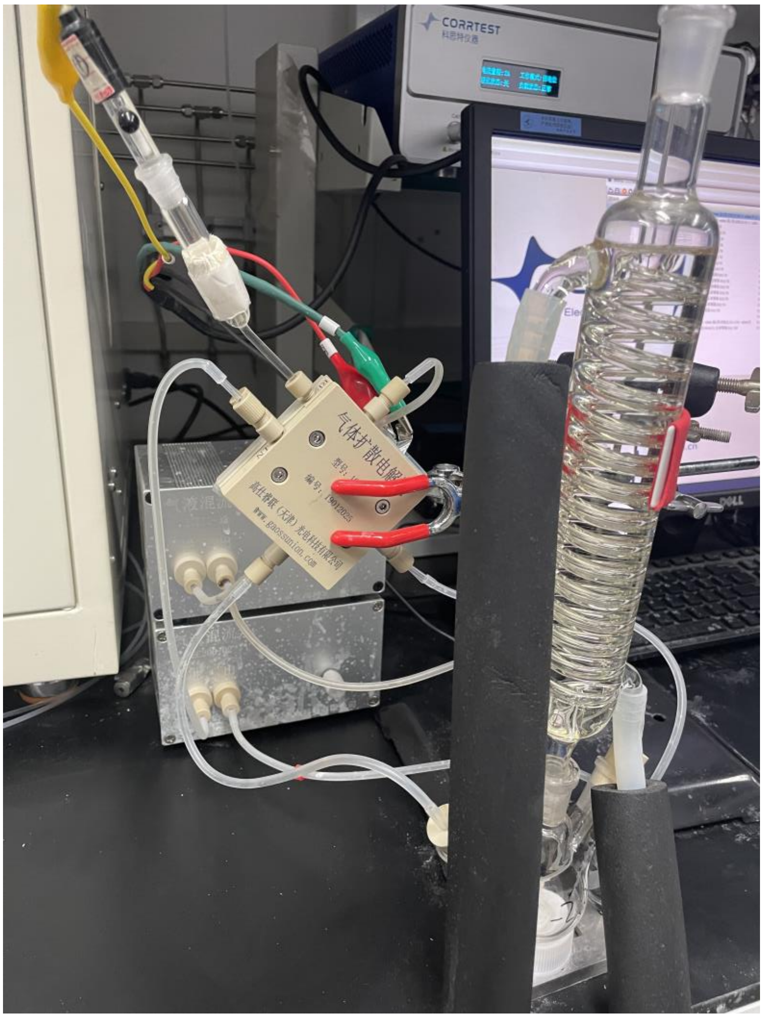

Supplementary Figure 1. The photograph of the used flow cell with an electrode area of 1 cm^2.

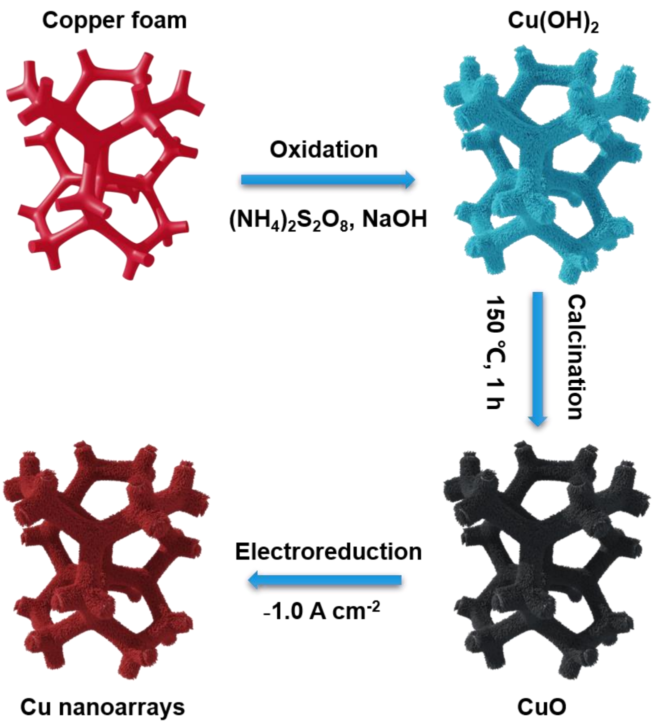

Supplementary Figure 2. Synthetic route for Cu NAs on the Cu foam.

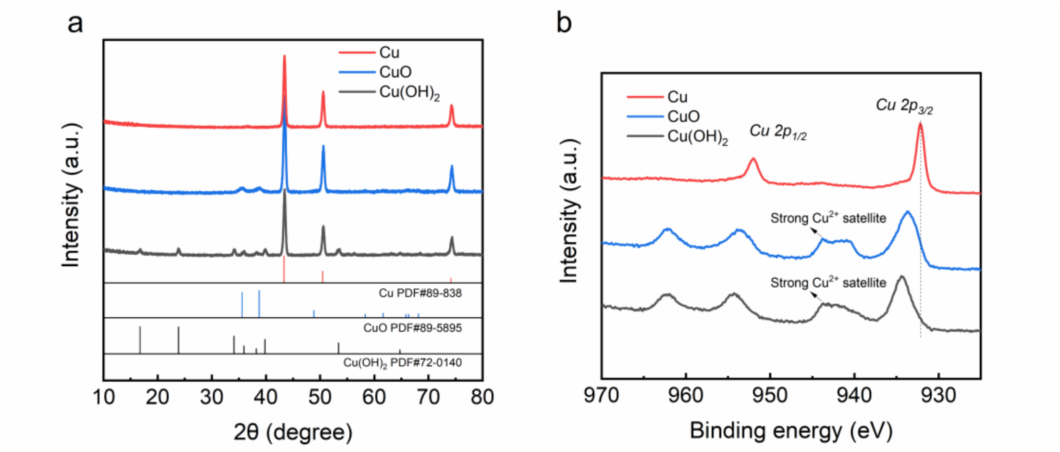

Supplementary Figure 3. Structural characterization of the catalysts. a, XRD patterns and **b**, XPS spectra of $Cu(OH)_2$, CuO and Cu NAs.

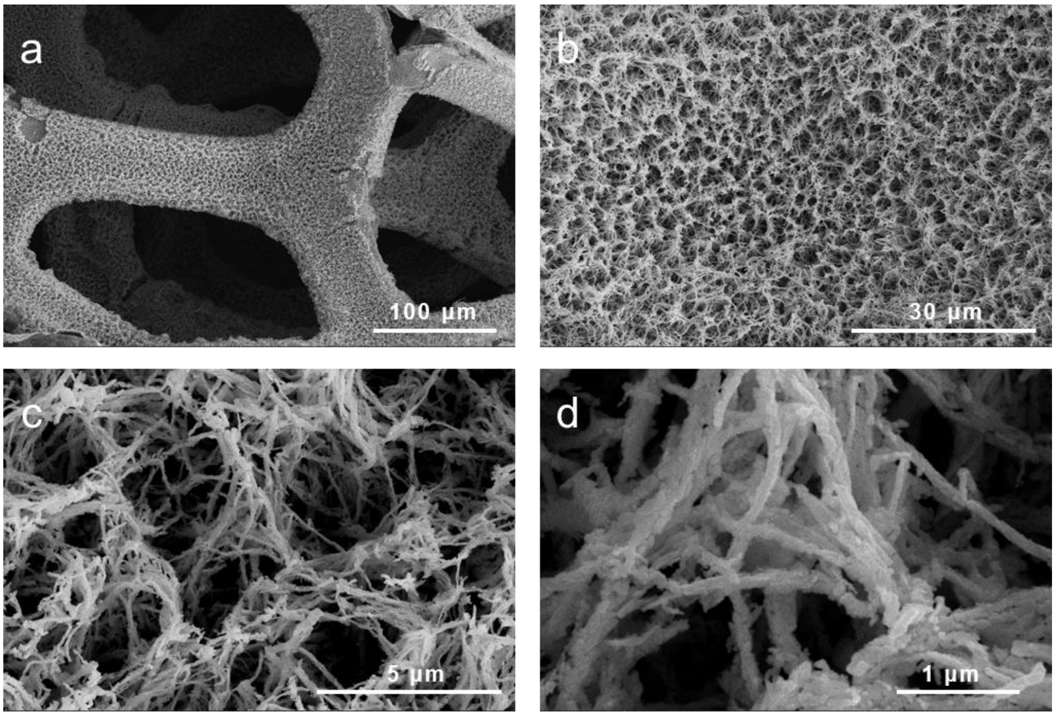

Supplementary Figure 4. Morphology of the Cu NAs on Cu foam. a-d, SEM images with different magnification.

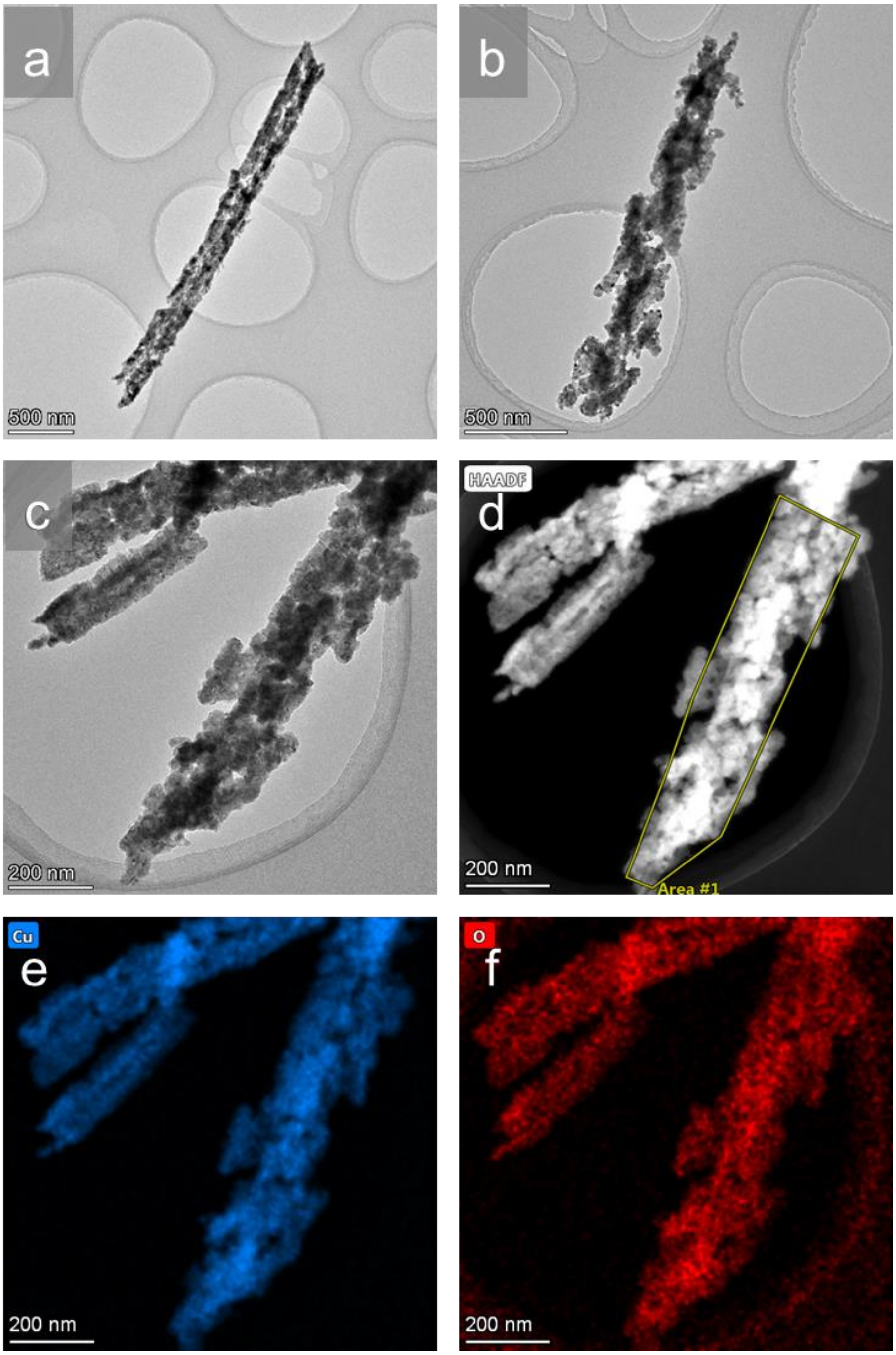

Supplementary Figure 5. TEM and HAADF images of Cu nanoarrays. a–c, TEM, and **d**, HAADF images of Cu NAs. The related elemental mapping images of Cu NAs: **e**, Cu and **f**, O elements.

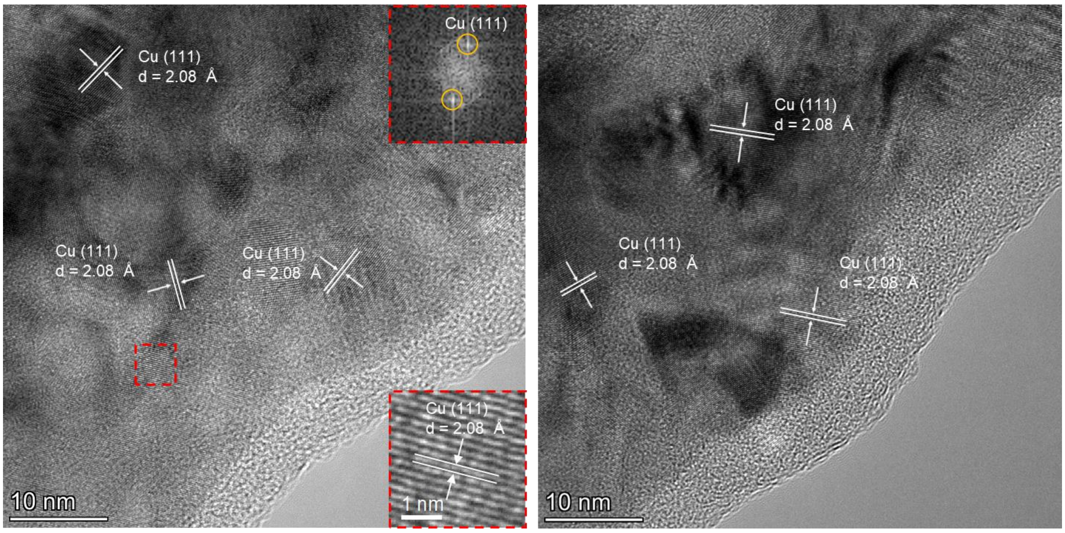

Supplementary Figure 6. HRTEM images of the as-prepared Cu NAs.

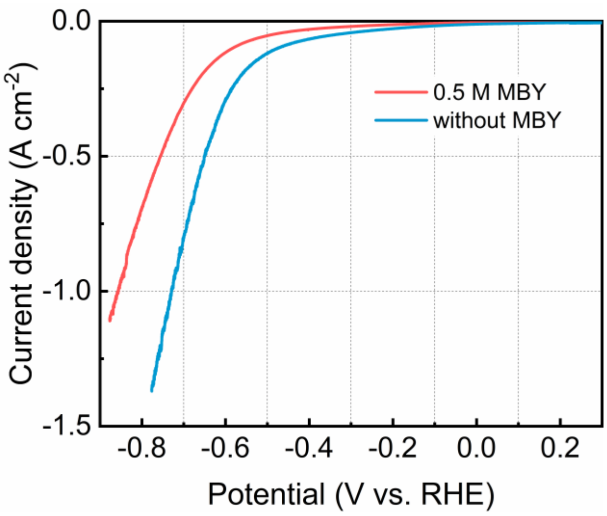

Supplementary Figure 7. The polarization curves of Cu NAs with and without MBY in 1 M KOH electrolyte.

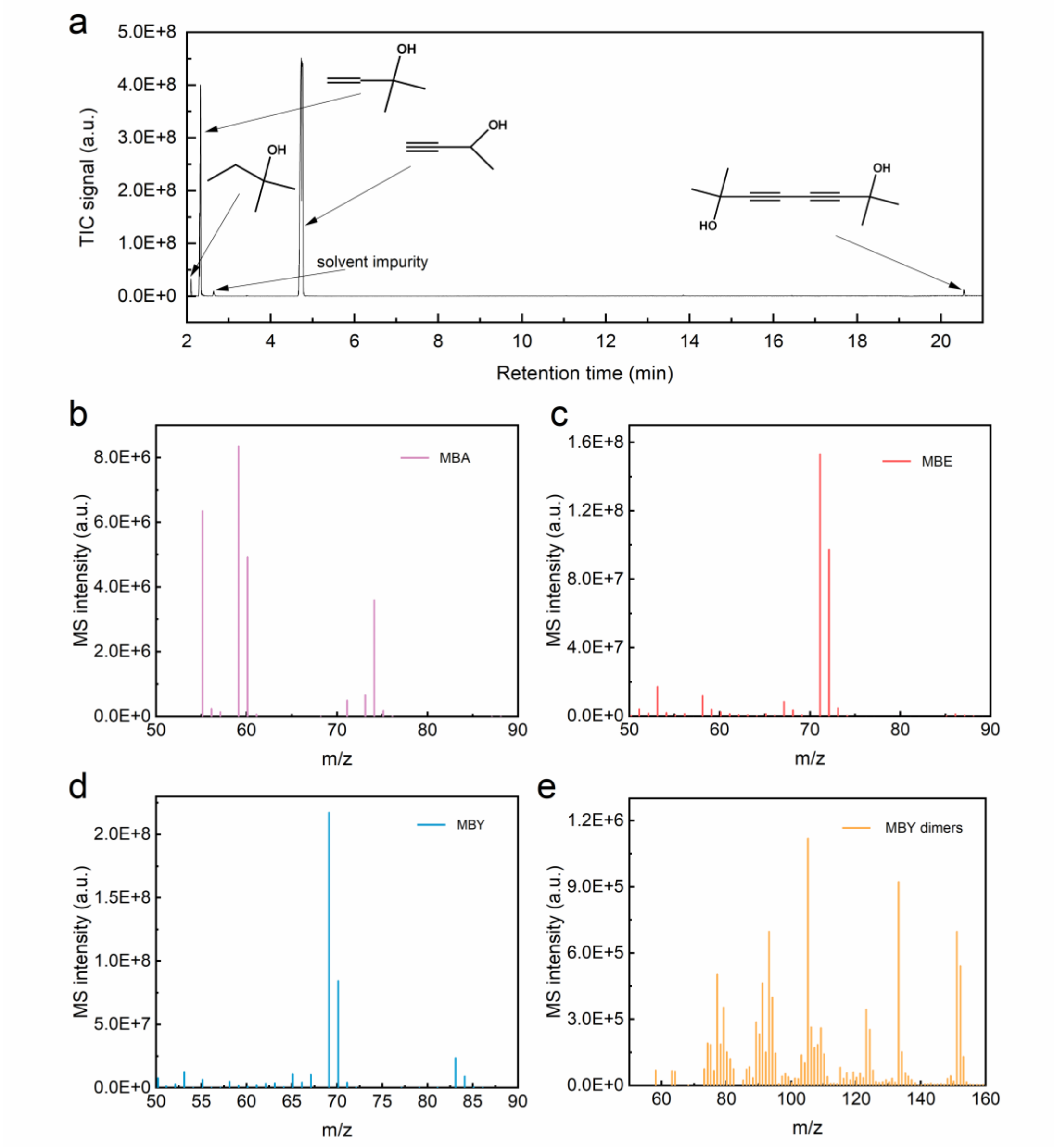

Supplementary Figure 8. GC-MS analysis of the liquid products after the electrocatalytic MBY hydrogenation. a, Total ion chromatogram (TIC) versus retention time. Data before two minutes was not acquired to avoid interference from solvent peaks. Mass fragmentation patterns acquired at different retention time: **b**, 2.1 min for MBA, **c**, 2.3 min for MBE, **d**, 4.7 min for MBY and **e**, 20.5 min for MBY dimers. **The above electrocatalytic MBY hydrogenation on Cu NAs was operated at −1.3 A cm^{-2} for 10 min in 1 M KOH aqueous solution containing 0.5 M MBY.**

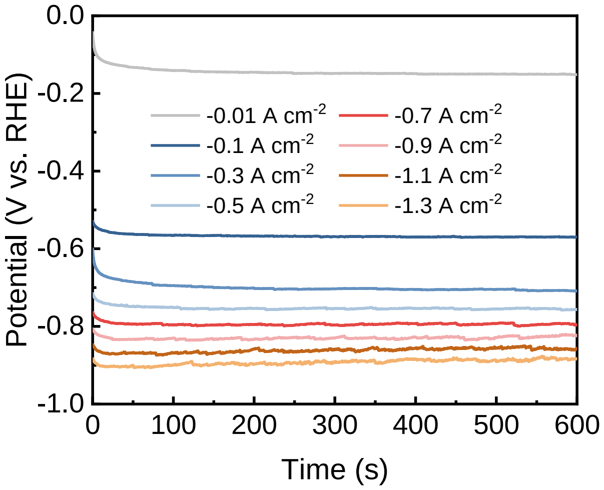

Supplementary Figure 9. Time dependent potentials during the Chronopotentiometry experiments of Cu NAs.

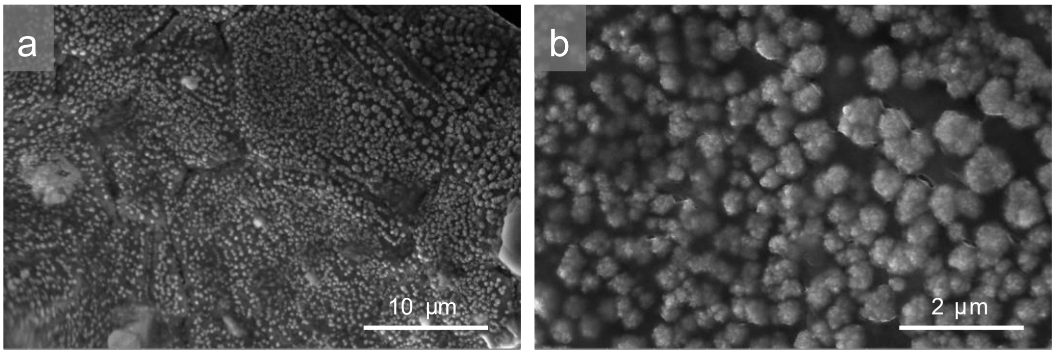

Supplementary Figure 10. Morphology of the Pd nanoparticles on Cu foam. a-b, SEM images with different magnification.

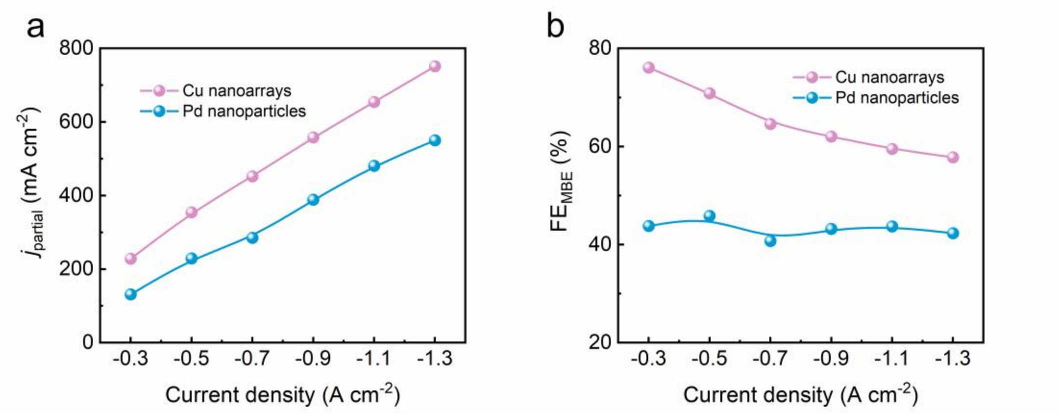

Supplementary Figure 11. The electrocatalytic MBY semi-hydrogenation performance of Cu NAs and Pd nanoparticles. **a**, partial current densities and **b**, Faradaic efficiency of MBE versus the applied current density.

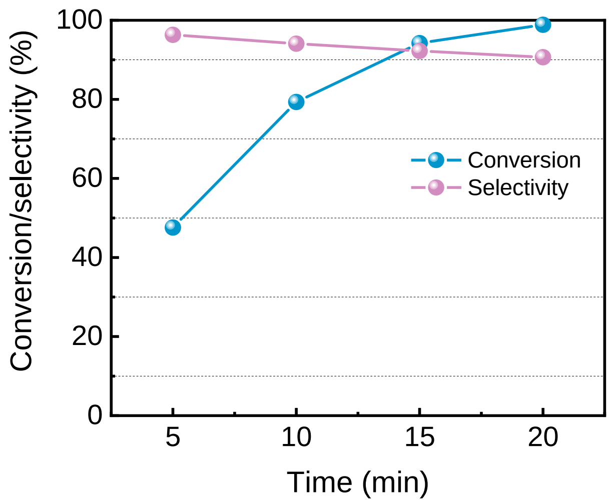

Supplementary Figure 12. Time dependent MBY conversion and MBE selectivity of Cu NAs at −1.3 A cm^{-2}.

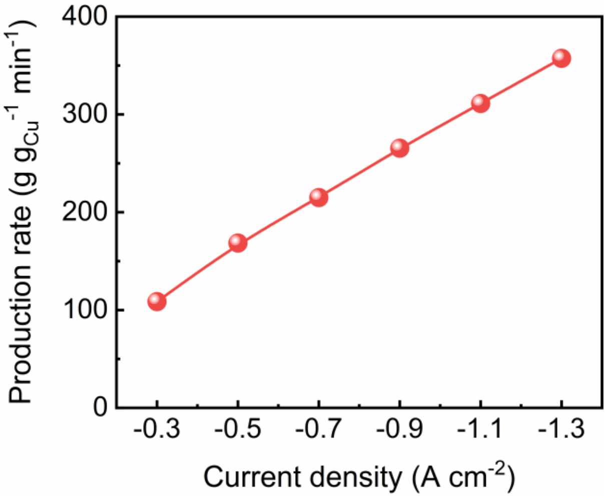

Supplementary Figure 13. The MBE production rate of Cu NAs versus the applied current density.

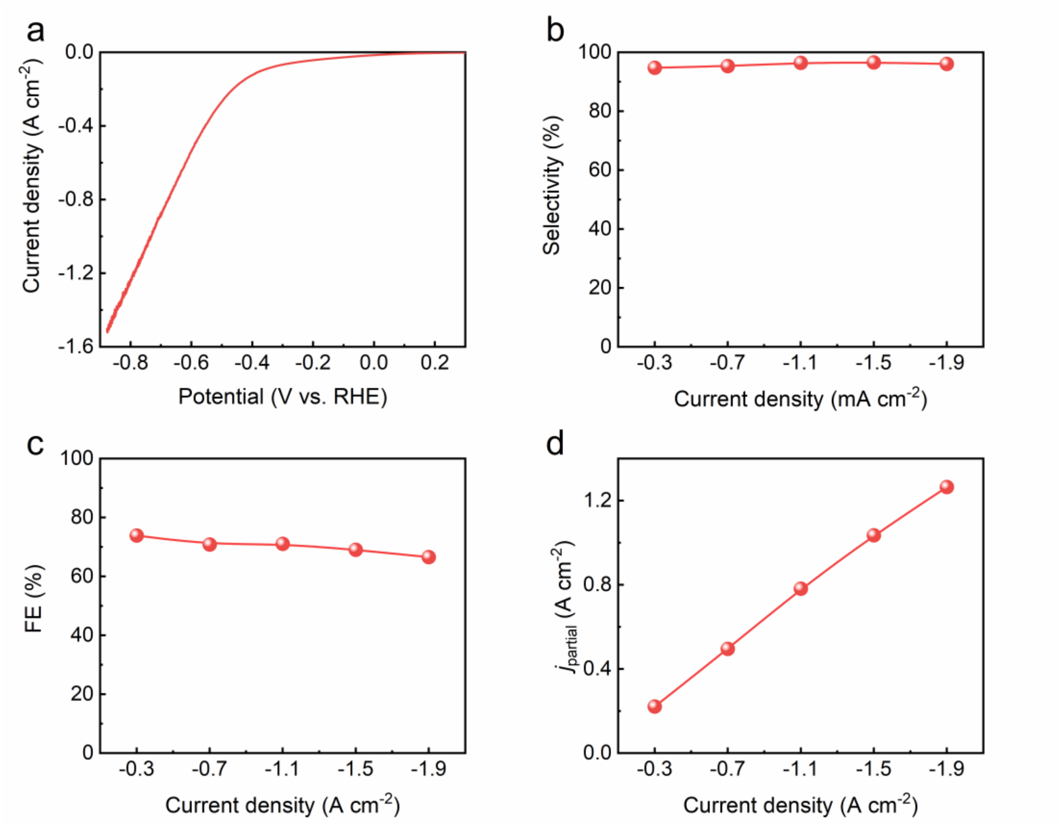

Supplementary Figure 14. The electrocatalytic MBY semi-hydrogenation performance of Cu NAs at 60 °C. a, LSV curves, **b**, selectivity, **c**, Faradaic efficiency and **d**, partial current densities of MBE versus the applied current density.

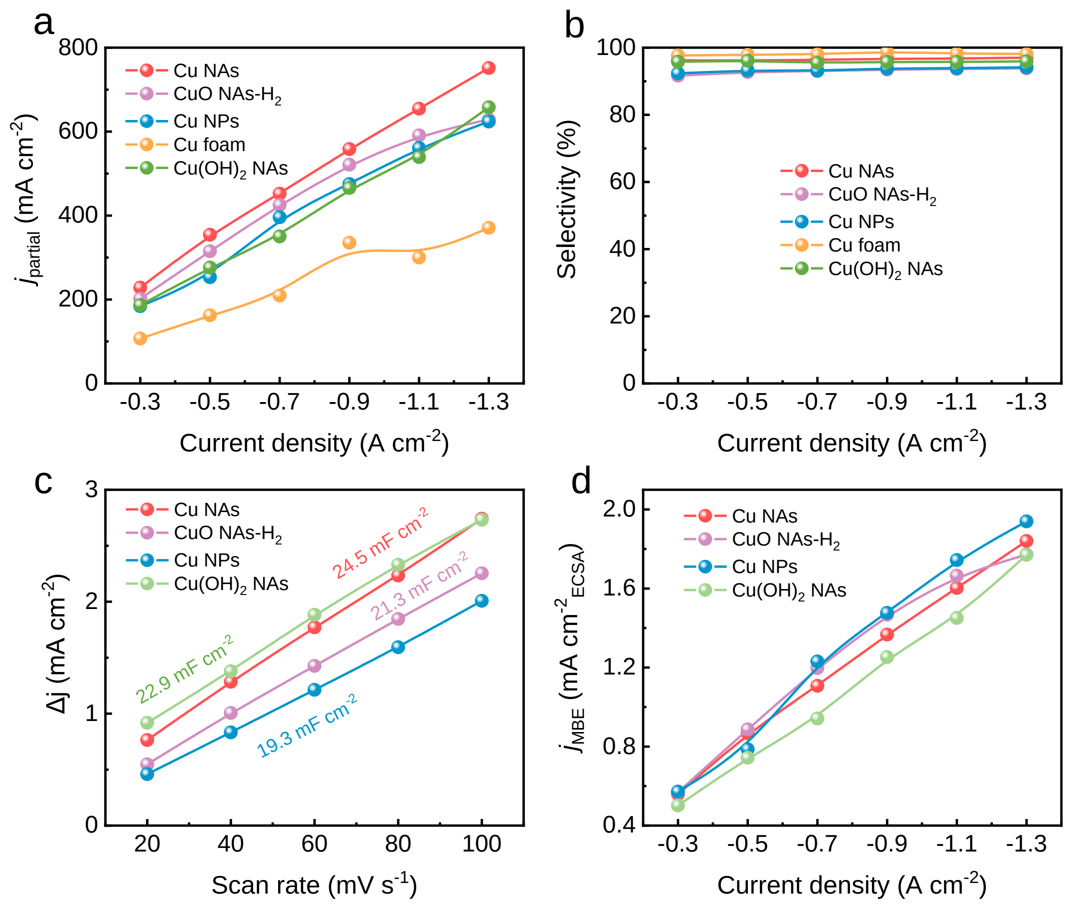

Supplementary Figure 15. The electrocatalytic MBY semi-hydrogenation performance on several Cu-based catalysts. a, The partial current density, **b,** specific selectivity of MBE, **c,** C_{dl} and **d,** ECSA normalized partial current density of MBE on different Cu catalysts in the 1 M KOH solution containing 0.5 M MBY.

Here, $Cu(OH)_2$ NAs was in-situ electrochemically reduced in 1 M KOH aqueous electrolyte at –1.0 A cm^{-2} for 10 min before the electrocatalytic evolution. The CuO NAs-H_2 was fabricated by annealing the $Cu(OH)_2$ nanoarrays at 150 ºC for 1 hour in air with subsequent reduction at 200 ºC for 1 hour in H_2. Cu NPs were synthesized through an electrodeposition approach. The deposition electrolyte contained 1 M KOH, 0.1 M copper nitrate and 0.2 M sodium tartrate dibasic dihydrate. The electrodeposition was conducted at −0.2 A cm^{-2} for 90 s on the copper foam.

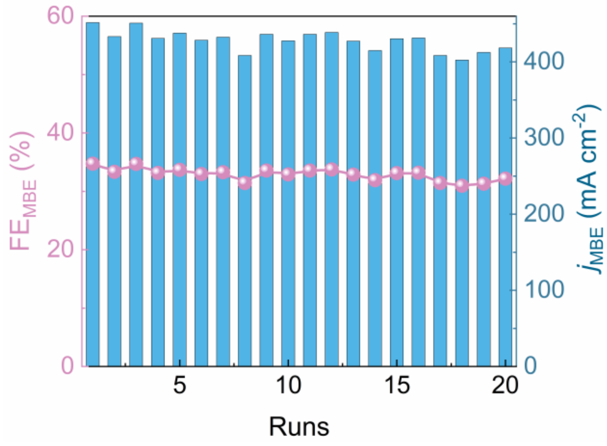

Supplementary Figure 16. The Faradaic efficiency and partial current density of MBE for each run during the recycling stability experiment.

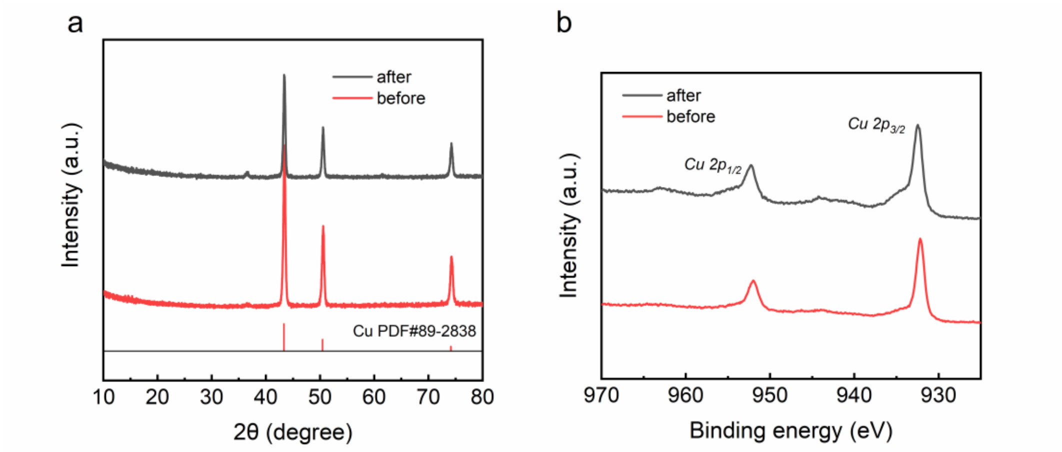

Supplementary Figure 17. Structural characterization of the catalysts. a, XRD and **b**, XPS patterns of the Cu NAs before and after 20 recycling runs.

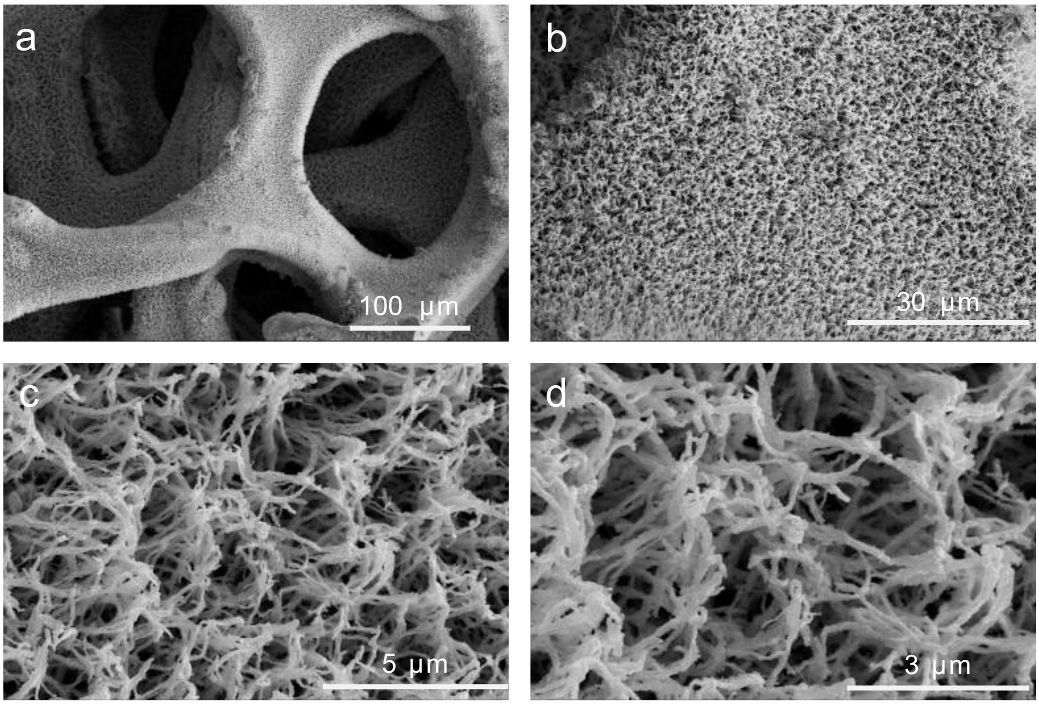

Supplementary Figure 18. Morphology of the Cu NAs after 20 recycling runs. **a-d,** SEM images with different magnification.

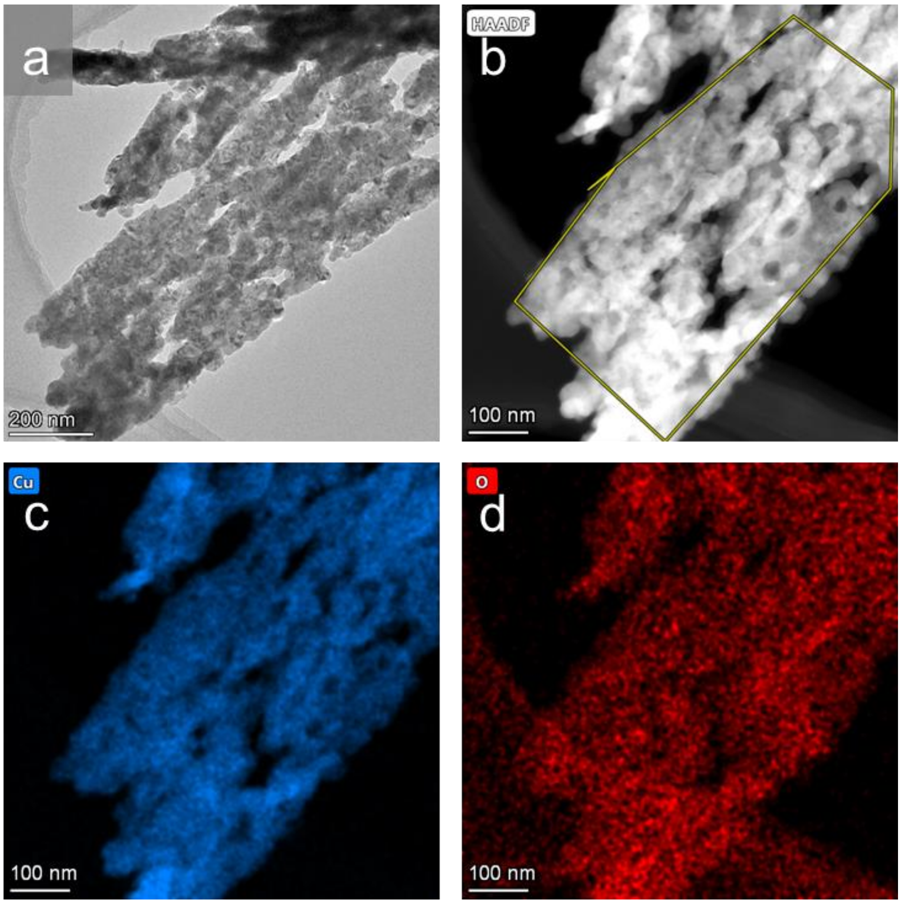

Supplementary Figure 19. TEM and HAADF images of Cu NAs after 20 recycling runs. a, TEM and **b**, HAADF images. The related elemental mapping images of the Cu NAs after 20 recycling runs: **c**, Cu and **d**, O elements.

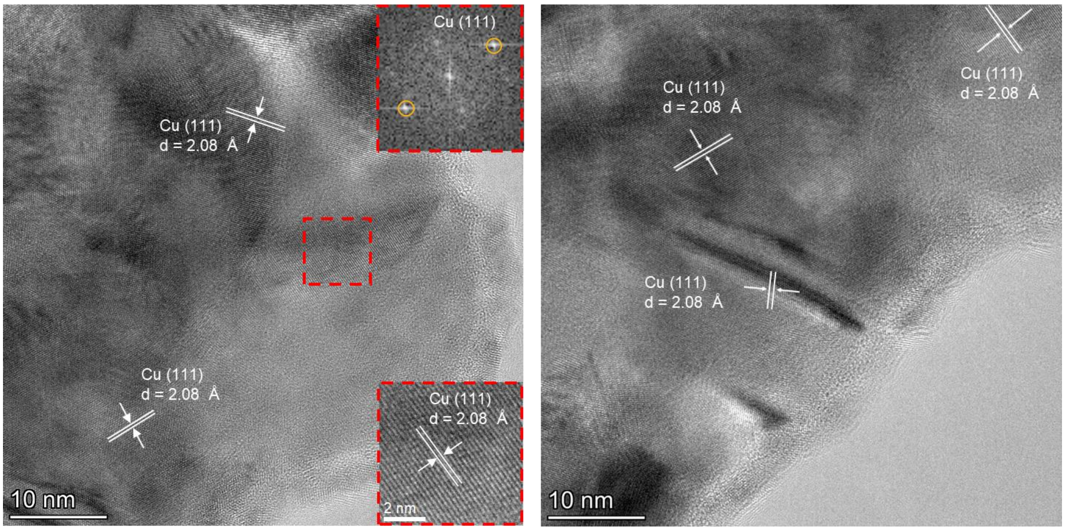

Supplementary Figure 20. HRTEM images of Cu NAs after 20 recycling runs.

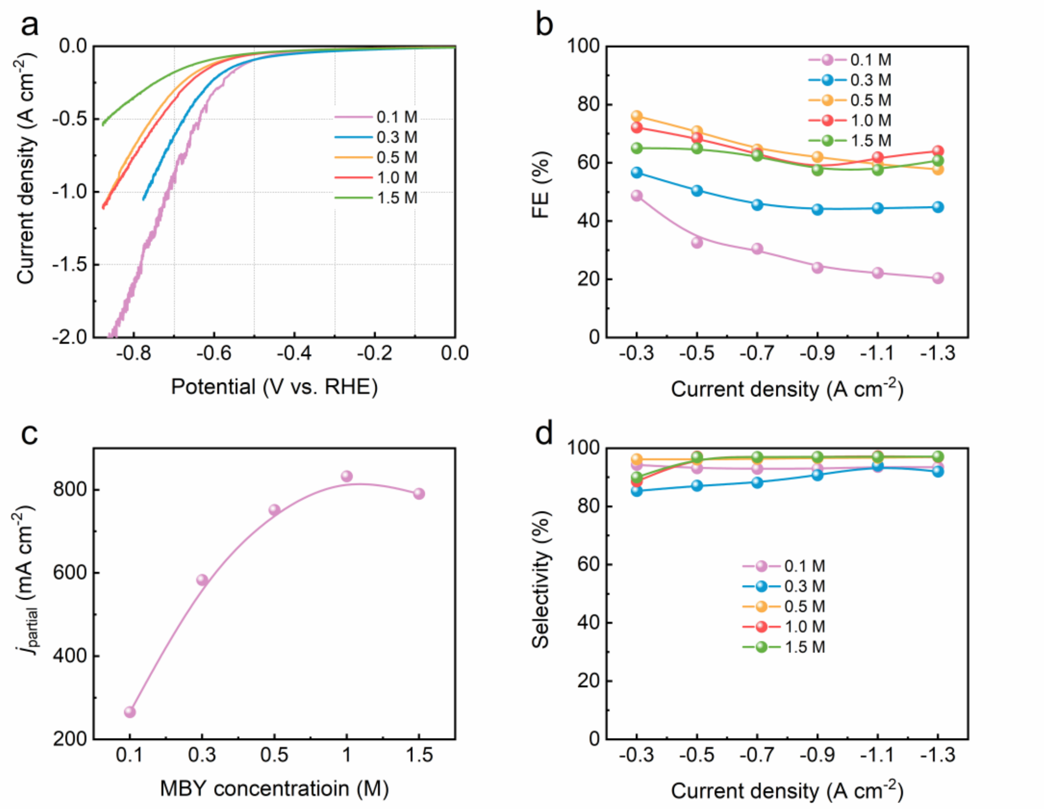

Supplementary Figure 21. The influence of MBY concentration on the electrocatalytic MBY semi-hydrogenation performance of Cu NAs. a, Polarization curves, **b**, Faradaic efficiency of MBE versus applied current density, **c**, partial current densities of MBE versus MBY concentration at –1.3 A cm^{-2}, and **d**, MBE selectivity versus applied current density.

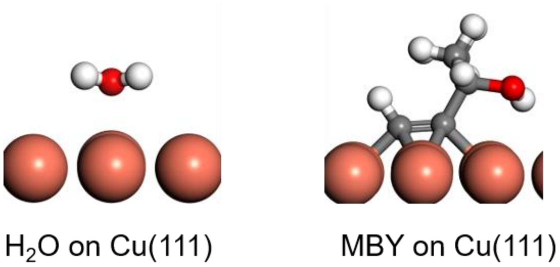

	E_{ads} (eV)
H_2O on Cu(111)	-0.33
MBY on Cu(111)	-3.20

Supplementary Figure 22. The adsorption energy of H_2O and MBY on Cu(111).

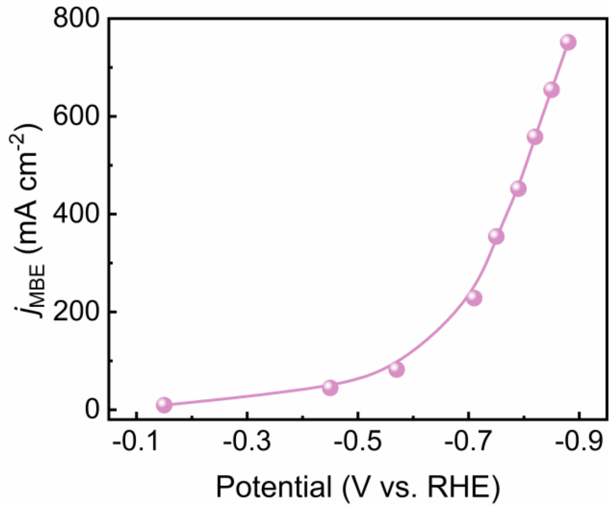

Supplementary Figure 23. The potential dependent partial current density of MBE. Cathode electrolyte: 20 mL 1 M KOH solution containing 0.5 M MBY.

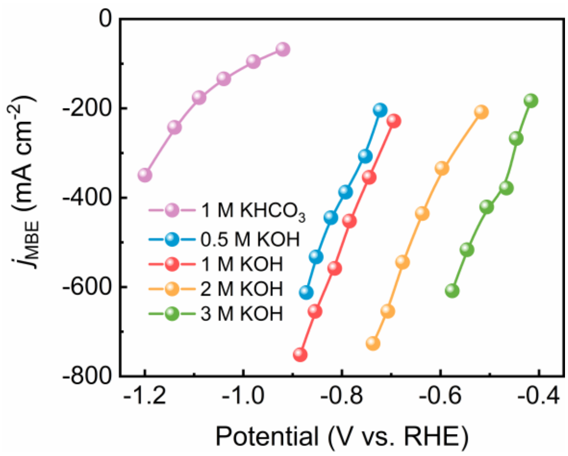

Supplementary Figure 24. The potential dependent partial current density of MBE at different pH values.

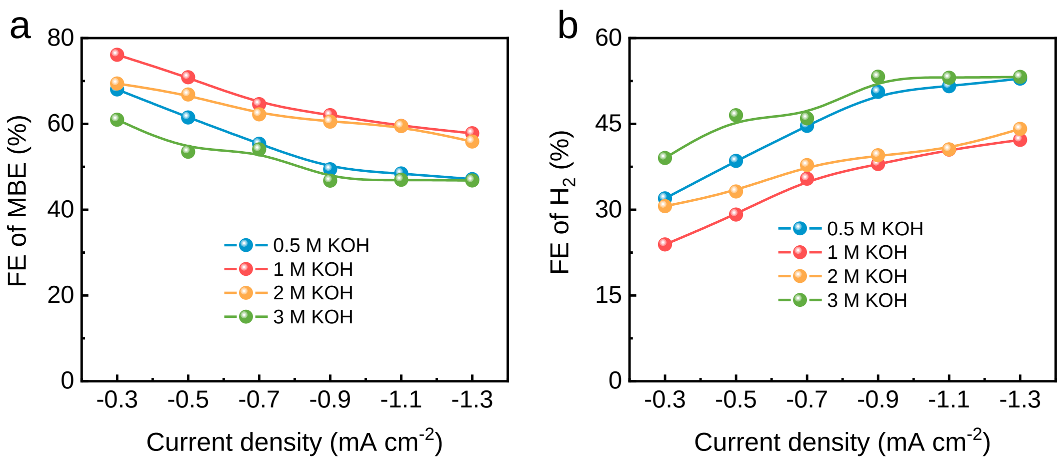

Supplementary Figure 25. The FE of **(a)** MBE and **(b)** H_2 in different alkaline electrolyte.

As shown in Supplementary Figure. 25 b, when the concentration of KOH electrolyte was changed from 0.5 M to 3 M, the FE of H_2 first decreased in 1 M KOH and then increased in 2 M and 3 M KOH solution. Clearly, along with increased pH values, the FE changes of MBE and H_2 was opposite, indicating their strong competition. Therefore, after the formation of surface H*, the competition between MBY hydrogenation and H_2 production occurred. Based the selectivity, the optimal concentration of KOH is 1 M.

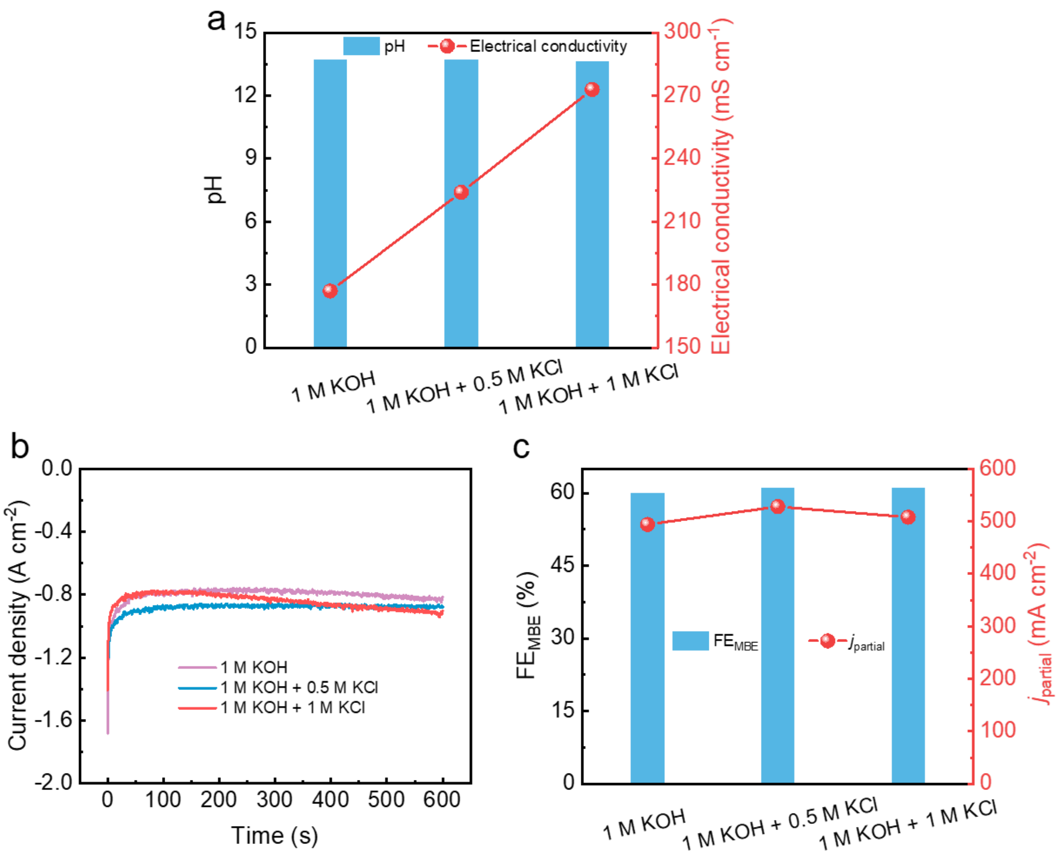

Supplementary Figure 26. The influence of electrolyte conductivity on MBY hydrogenation. a, The pH values and electrical conductivity of the KOH electrolyte without and with KCl. **b,** Current density profiles for MBY hydrogenation on Cu NAs in different electrolytes. **c,** The FE and j_{partial} of MBE in different electrolytes. Reaction conditions: −0.8 V vs. RHE; 0.5 M MBY in electrolyte.

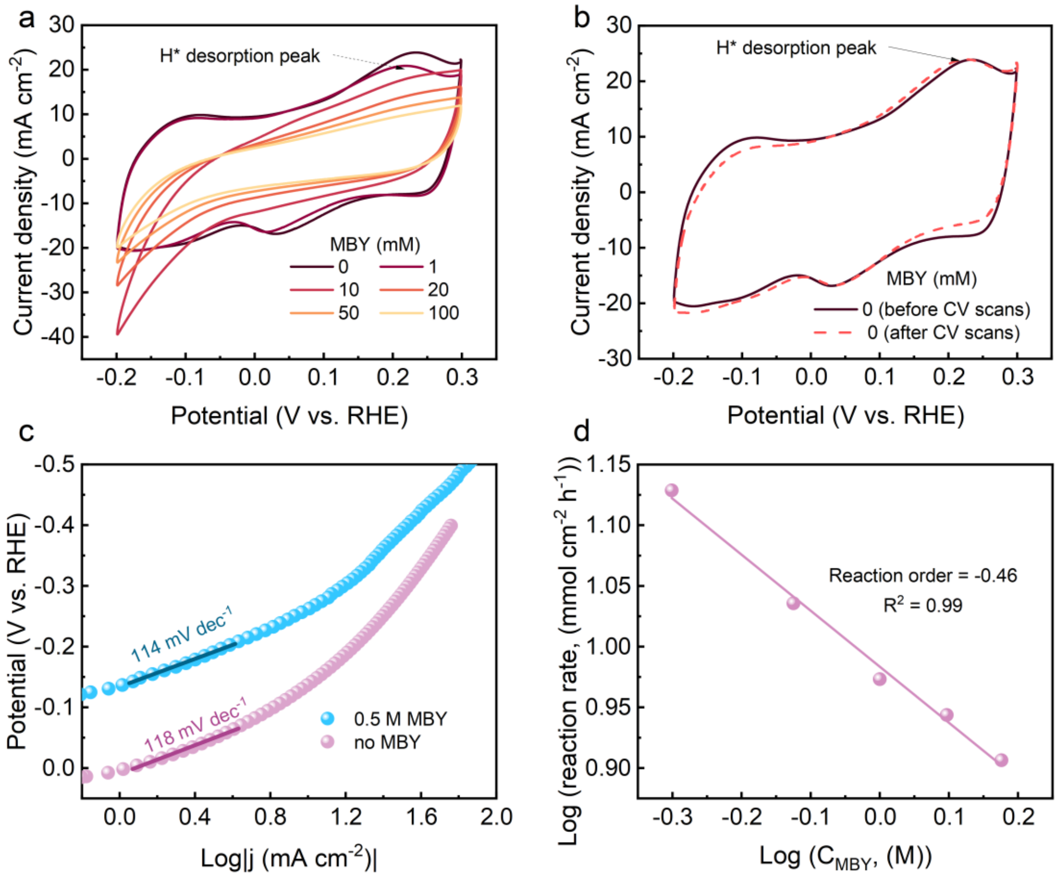

Supplementary Figure 27. Mechanism and kinetics of electrochemical MBY hydrogenation. a, CV curves of Cu NAs in 1 M KOH solution containing different concentrations of MBY. **b,** CV curves of Cu NAs in KOH electrolyte before and after CV scans in KOH electrolytes containing MBY. **c,** Tafel plots of Cu NAs in KOH electrolyte with and without MBY. **d,** The electrocatalytic productivity of MBE over Cu NAs as a function of MBY concentration in 1 M KOH electrolyte. Reaction conditions: −0.9 V vs. RHE; the MBY conversion of <20%.

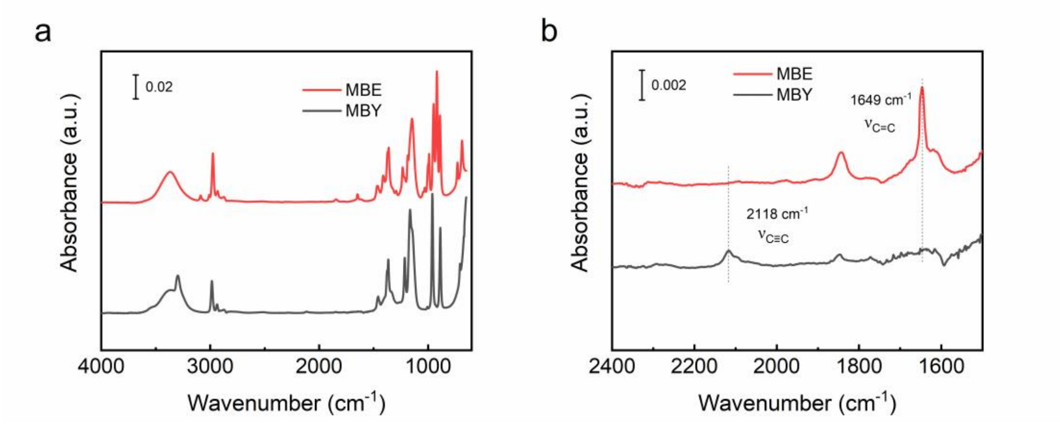

Supplementary Figure 28. a, IR spectra of MBY and MBE in liquid phase. **b,** the enlarged image between 1500 to 2400 cm^{-1}.

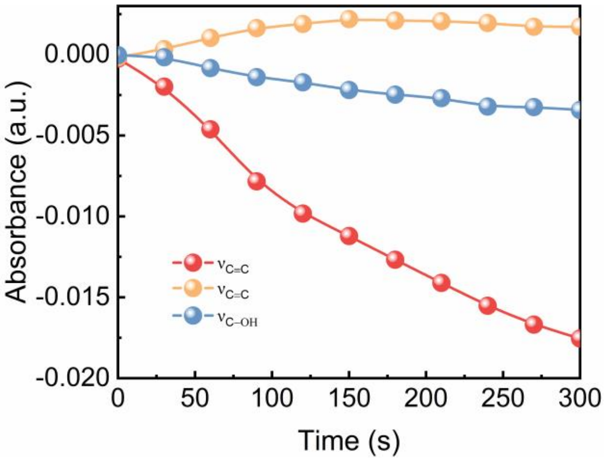

Supplementary Figure 29. The peak intensities of carbon-carbon triple bonds, carbon-carbon double bonds, and C–OH bonds versus reaction time.

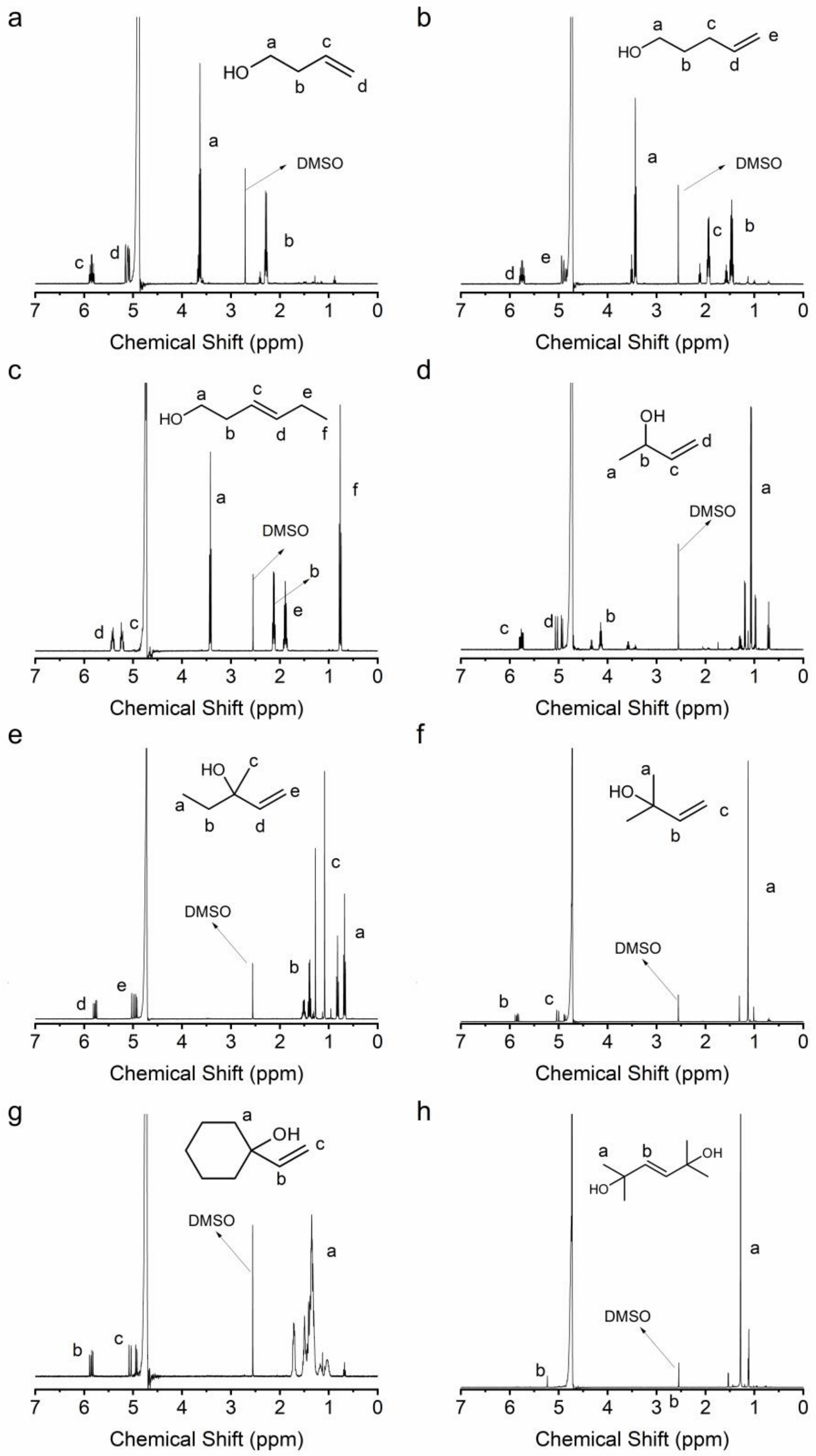

Supplementary Figure 30. ^1H NMR spectra of the corresponding alkenols. a, 3-Buten-1-ol, **b**, 4-penten-1-ol, **c**, 3-hexene-1-ol, **d**, 3-buten-2-ol, **e**, 3-methyl-1-penten-3-ol, **f**, 2-methyl-3-butene-2-ol, **g**, 1-ethenylcyclohexanol and **h**, 2,5-dimethyl-3-hexene-2,5-diol.

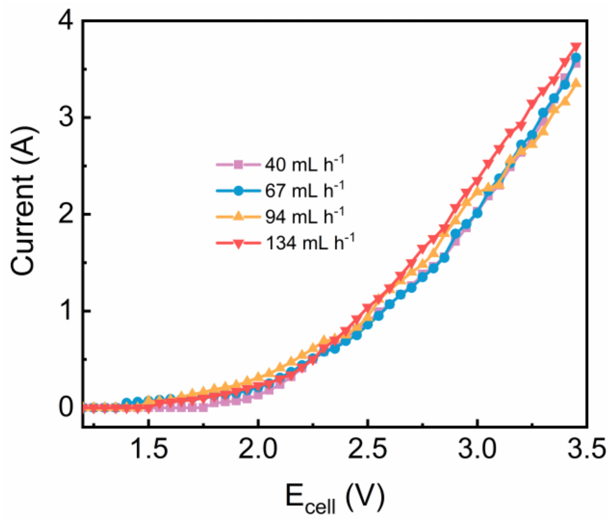

Supplementary Figure 31. The LSV curves of Cu NAs at different flow rates of the electrolyte.

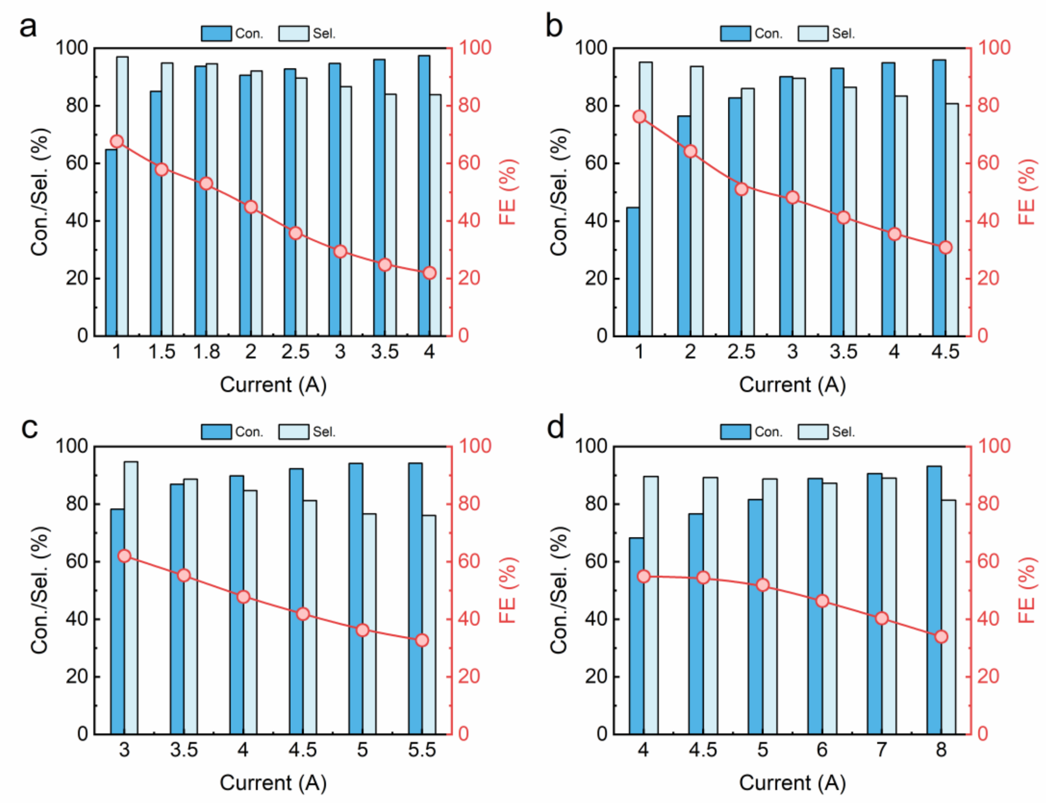

Supplementary Figure 32. The applied current-dependent MBY single-pass conversion, MBE selectivity and Faradaic efficiency of Cu NAs at different electrolyte flow rates: a, 40, **b**, 67, **c**, 94, and **d**, 134 mL h^{-1}.

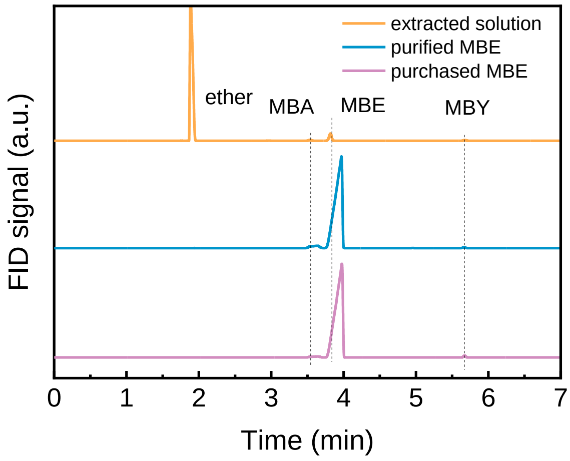

Supplementary Figure 33. Gas chromatography curves of the samples before and after purification by preparative chromatography and standard sample purchased from Shanghai Macklin Biochemical Co., Ltd.

We performed isolation of the products from the electrolytic mixture (MBY conversion = 91%, MBE selectivity = 93%). The liquid products were firstly extracted with ether from the 1 M KOH electrolyte, then purified by distillation and subsequent preparative chromatography. The samples were quantified by gas chromatography (Fuli GC9790Plus) equipped with a flame ionization detector (FID) (Supplementary Fig. 33). Finally, the purity of MBE reached to as high as 98% by our approach, which was same with the commercial MBE (98%) e.g., Sigma Aldrich (98%), Shanghai Macklin Biochemical Co., Ltd (98%).

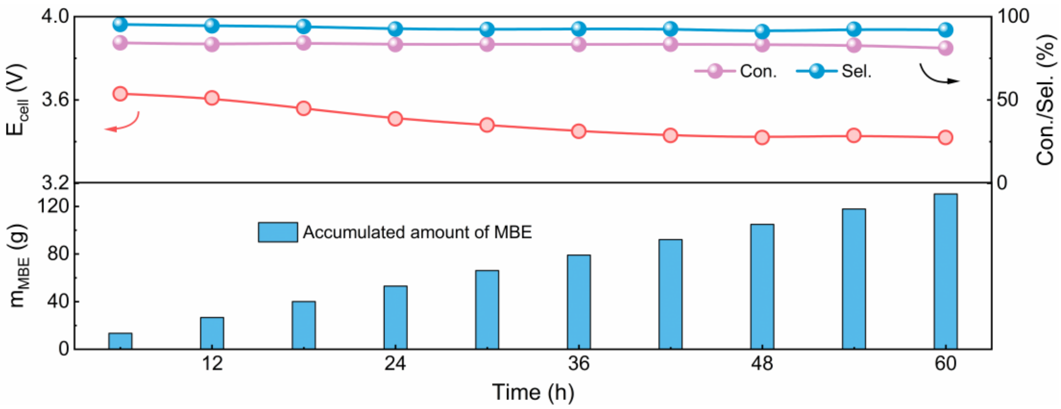

Supplementary Figure 34. Continuous 60-hour production of MBE at a cell current of 3.0 A by feeding 1 M KOH aqueous solution containing 0.5 M MBY with a flow rate of 67 mL h^{-1}.

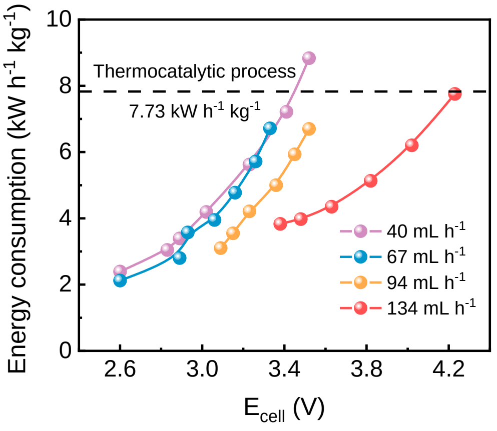

Supplementary Figure 35. The energy consumption of 1 kg MBE at different cell voltages and electrolyte flow rates.

As shown in Supplementary Figure 35, we compare the consumed energy for producing 1 kg MBE using electrocatalytic and thermocatalytic processes. The energy consumption of the thermocatalytic MBE production is estimated to be 7.73 kW h^{-1} kg^{-1} (950 kgce/t) according to similar energy consumption of butynediol hydrogenation. The energy consumption of electrocatalytic MBE production mainly changes from 2 to 6 kW h^{-1} kg^{-1} under different conditions, which is substantially lower than 7.73 kW h^{-1} kg^{-1} for the thermocatalytic process. Especially, for continuous 8-hour electrochemical production of MBE at a cell current of 1.8 A ($E_{cell} \approx 2.89$ V), the energy consumption is only 3.39 kW h^{-1} kg^{-1}, which is about 44% of those for the thermocatalytic process. Therefore, the electrocatalytic approach is more energy-efficient than the thermocatalytic process.

Supplementary Table 1. The comparison of MBY semi-hydrogenation performance between electrocatalytic and thermocatalytic processes.

Catalysts	Production rate (g_{MBE} g_{cat}^{-1} h^{-1})	Conversion (%)	Selectivity (%)	Stability (h)	H_2 pressure (bar)	Temperature (°C)	Reference
**Cu nanoarrays**	**357**	**96**	**97**	**20**	**0**	**25**	**this work** (three electrode) (1.3 A cm^{-2})
**Cu nanoarrays**	**169**	**91**	**90**	–	**0**	**25**	**this work** (two electrode) (280 mA cm^{-2})
**Cu nanoarrays**	**56**	**91**	**93**	**8**	**0**	**25**	**this work** (two electrode) (72 mA cm^{-2})
Pd(B,C)	16	100	95	–	1	25	*Nat. Commun.* ***13****, 2754 (2022)*
Pd/ZnO	19	99	94	–	5	35	*J. Catal 251, 213-222 (2007)*
PdZn/CN@ZnO	9	92	95	–	5	35	*J. Catal 350, 13-20 (2017)*
Pd-In/In_2O_3	4	99	95	10	1	80	*Green Chem. 21, 4143-4151 (2019)*
Pd/TiO_2-V_O@C	88	99	93	9	2	25	*ACS Catal. 9, 10656-10667 (2019)*
Pd-DETA	49	99	95	–	1	35	*ACS Appl. Mater. Interfaces 13, 31775-31784 (2021)*

Supplementary Note 1. Techno-economic assessment.

To determine the economic potential of renewable electricity powered production of MBE from MBY semi-hydrogenation, we conducted a techno-economic analysis (TEA) based on a modified model from previous work[1,2].

Below is the list of assumptions made for calculations.

1. The production capacity of the plants is 1 ton of MBE per day.
2. The total catalyst and membrane cost is 5 % of the total electrolyzer cost.
3. The total cost of the electrolyzer is $10,000 per m^2.
4. The price of electricity is 5 ¢/kWh.
5. The separation cost is assumed to be 20% of the electricity cost.
6. Other operation costs are assumed to be 10% of the electricity cost.
7. The lifetime of the electrolyzer is 1 year.
8. The Faradaic efficiency to MBE is 60%, 50%, 40% and 30%, when the cell operating voltage is 3.0 V, 3.5 V, 4.0 V and 4.5 V, and the total operating current density is 100 mA/cm^{-2}, 200 mA/cm^{-2}, 300 mA/cm^{-2} and 400 mA/cm^{-2}, respectively.
9. The prices of MBY and MBE are assumed to be $30,000 per ton and $60,000 per ton respectively.

Supplementary References:

1. Li, Y. *et al.* Redox-mediated electrosynthesis of ethylene oxide from CO2 and water. *Nat. Catal.*, 1-8 (2022).
2. Leow, W. R. *et al.* Chloride-mediated selective electrosynthesis of ethylene and propylene oxides at high current density. *Science* **368**, 1228-1233 (2020).
